# Application of zinc chloride precipitation method for rapid isolation and concentration of infectious *Pectobacterium* spp. and *Dickeya* spp. lytic bacteriophages from surface water and plant and soil extracts

**DOI:** 10.1007/s12223-015-0411-1

**Published:** 2015-06-24

**Authors:** Robert Czajkowski, Zofia Ozymko, Ewa Lojkowska

**Affiliations:** Department of Biotechnology, Intercollegiate Faculty of Biotechnology, University of Gdansk and Medical University of Gdansk, Kladki 24, 80-822 Gdansk, Poland

## Abstract

This is the first report describing precipitation of bacteriophage particles with zinc chloride as a method of choice to isolate infectious lytic bacteriophages against *Pectobacterium* spp. and *Dickeya* spp. from environmental samples. The isolated bacteriophages are ready to use to study various (ecological) aspects of bacteria-bacteriophage interactions. The method comprises the well-known precipitation of phages from aqueous extracts of the test material by addition of ZnCl_2_, resuscitation of bacteriophage particles in Ringer’s buffer to remove the ZnCl_2_ excess and a soft agar overlay assay with the host bacterium to isolate infectious individual phage plaques. The method requires neither an enrichment step nor other steps (e. g., PEG precipitation, ultrafiltration, or ultracentrifugation) commonly used in other procedures and results in isolation of active viable bacteriophage particles.

## Introduction

Soft rot *Enterobacteriaceae* (SRE): *Pectobacterium* spp. and *Dickeya* spp. are necrotrophic plant pathogenic bacteria able to cause disease symptoms on a large number of monocot and dicot plants worldwide (Gardan et al. 2003; Perombelon 1991; Toth et al. 2011). Although they are not recognized as quarantine bacteria in Europe, they can cause up to 50 % crop reduction resulting in significant economic losses. There is currently no effective disease control method apart from reliance on seed certification and crop production hygiene. An ecologically friendly effective method to control diseases caused by these bacteria may be one based on the use of lytic bacteriophages isolated from the agricultural environments (Czajkowski et al. 2013).

Bacteriophages (phages) are viruses that can specifically infect and lyse bacterial cells (Abedon 2009; d’Herelle 1930; Hadley 1928). They were independently discovered and described for the first time at the beginning of the twentieth century by Frederick W. Twort in England in 1915 and by Felix d’Herelle in France in 1917 as filterable, transmissible agents provoking bacterial lysis. Phages are believed to be the most abundant biological forms in nature, with numbers ranging from 10^8^ to 10^14^ plaque forming units (PFU) per gram of soil and/or milliliter of water (Ashelford et al. 2003). Ecologically, bacteriophages are present virtually everywhere and are as diverse as their bacterial hosts, being able to survive under extreme conditions of high and low temperature and very low or very high pH (Abedon 2009).

Lytic bacteriophages have been proposed to control bacterial infections in plants. They have been evaluated against different plant pathogens viz. *Erwinia amylovora*, *Xanthomonas pruni*, *Pseudomonas tolaasii*, *Streptomyces scabies* and *Ralstonia solanacearum* (for review see Jones et al. 2008). Bacteriophages were also tested (under laboratory conditions) to control pectinolytic *Pectobacterium* spp. and *Dickeya* spp. (Adriaenssens et al. 2012; Czajkowski et al. 2013, 2015). In our previous studies, we have isolated and characterized lytic bacteriophages infecting *Dickeya* spp. (Czajkowski et al. 2013) and broad host lytic bacteriophages able to infect *Dickeya* spp., *Pectobacterium wasabiae*, and *Pectobacterium carotovorum* subsp. *carotovorum* isolates (Czajkowski et al. 2015). These bacteriophages were assessed in detail for features important for the biological control activity (stability under different conditions of pH, temperature, UV radiation, and osmolarity) as well as evaluated in the proof-of-concept experiments as biocontrol agents against pectinolytic bacteria on potato tubers. The obtained results suggested that these bacteriophages would be valuable biological control agents under natural field conditions and during potato tuber storage.

The majority of studies on bacteriophages require the isolation of diverse, new phage particles from soil, water, sewage, and/or animal and plant samples. The standard method of bacteriophage isolation, so-called enrichment, is to incubate fresh cultures of the target bacteria with an inoculum which is expected to contain bacteriophages of interest (Wommack et al. 2008). After incubation, bacterial cells are removed by centrifugation and resulting supernatant assayed for phages by dilution plating in an overlay agar assay with the host bacterium. There are several drawbacks of the enrichment procedure; the method is not quantitative and therefore gives no indication of the original phage densities in the environment. In addition, the inoculum size is crucial to enrich phages in the host cultures, and if two or more different phages are present in the same sample, one can predominate the enrichment at the expense of the other.

Until now, a number of procedures have been developed to concentrate phages in order to avoid the enrichment in host bacterial cultures (Seeley and Primrose 1982; Wommack et al. 2008). Concentration procedures allow direct quantification of initial phage particles, and they do not select against rare or less vigorous viruses if several are present in one inoculum (Twest and Kropinski 2009). Various methods to concentrate phage particles have been evaluated under different conditions and for diverse applications, i. e., ultracentrifugation, ultrafiltration, dialysis, and adsorption to filters and chemicals (Seeley and Primrose 1982). These methods, although useful, are often laborious; some additionally require access to specific laboratory equipment and presence of the skillful personnel.

In 1991, Santos proposed to use zinc chloride for the rapid extraction of bacteriophage DNA and since then the method is used to purify bacteriophage genomic DNA worldwide (Santos 1991). The aim of this study was to adapt the well-known ZnCl_2_ precipitation method, used so far to purify bacteriophage genomic DNA (Santos 1991) only, to isolate and concentrate viable bacteriophage particles from environmental samples omitting the enrichment step in host bacterial cultures. To our best recollection, the ZnCl_2_ precipitation has never been used before to purify intact phage particles from environmental samples in order to use them later for epidemiological and ecological studies. Likewise, this method has never been used before to purify lytic bacteriophages against important plant pathogenic bacteria—*Pectobacterium* spp. and *Dickeya* spp. We believe that the application of zinc chloride precipitation for this purpose may help in obtaining new isolates of lytic bacteriophages *Dickeya* spp. and *Pectobacterium* spp. that may as yet remain unnoticed.

## Materials and methods

Phage ϕD5 (Czajkowski et al. 2013, 2014) was used in all experiments requiring spiking. The phage was propagated in its bacterial host *Dickeya solani* IPO2222 (van der Wolf et al. 2014).

*Pectobacterium* and *Dickeya* spp. isolates (*D. solani* IPO2222, *P. carotovorum* subsp. *carotovorum* Ecc71 and *Pectobacterium atrosepticum* SCRI1043) were grown at 28 °C for 24–48 h on tryptone soya agar (TSA, Oxoid) prior to use, unless stated otherwise. For liquid preparations, bacterial cultures were grown in tryptone soya broth (TSB, Oxoid) at 28 °C with agitation at 200 rpm.

Soil, stem, and potato tuber extracts were prepared in quarter-strength Ringer’s buffer (1/4 Ringer’s buffer) (Merck) as previously described (Czajkowski et al. 2010).

To test the efficiency of ZnCl_2_-based precipitation of bacteriophage particles and to compare this method with enrichment of bacteriophages in their host cultures (Twest and Kropinski 2009), 10 mL of sterile free from bacteriophages potato tuber extract, soil extract, TSB, or 1/4 Ringer’s buffer was spiked with ϕD5 phage suspension in water to obtain 1, 10, 100, and 1000 PFU/mL final concentration.

For enrichment of bacteriophages in the host bacterial cultures, 1 mL of ϕD5 phage suspensions containing 1, 10, 100, or 1000 PFU/mL in potato tuber extract, potato stem extract, soil extract, surface water, TSB, and 1/4 Ringer’s buffer were added to 9 mL of log-phase growing *D. solani* IPO2222 culture in TSB containing ca. 10^8^ colony forming units (CFU)/mL of bacteria and incubated overnight (ca. 16 h) at 28 °C with agitation (160 rpm). After this time, 100 μL of filter-sterilized (0.22 μm syringe filter) bacterial supernatant was assessed for phage presence with soft top agar method as described earlier (Czajkowski et al. 2013).

**Fig. 1:**
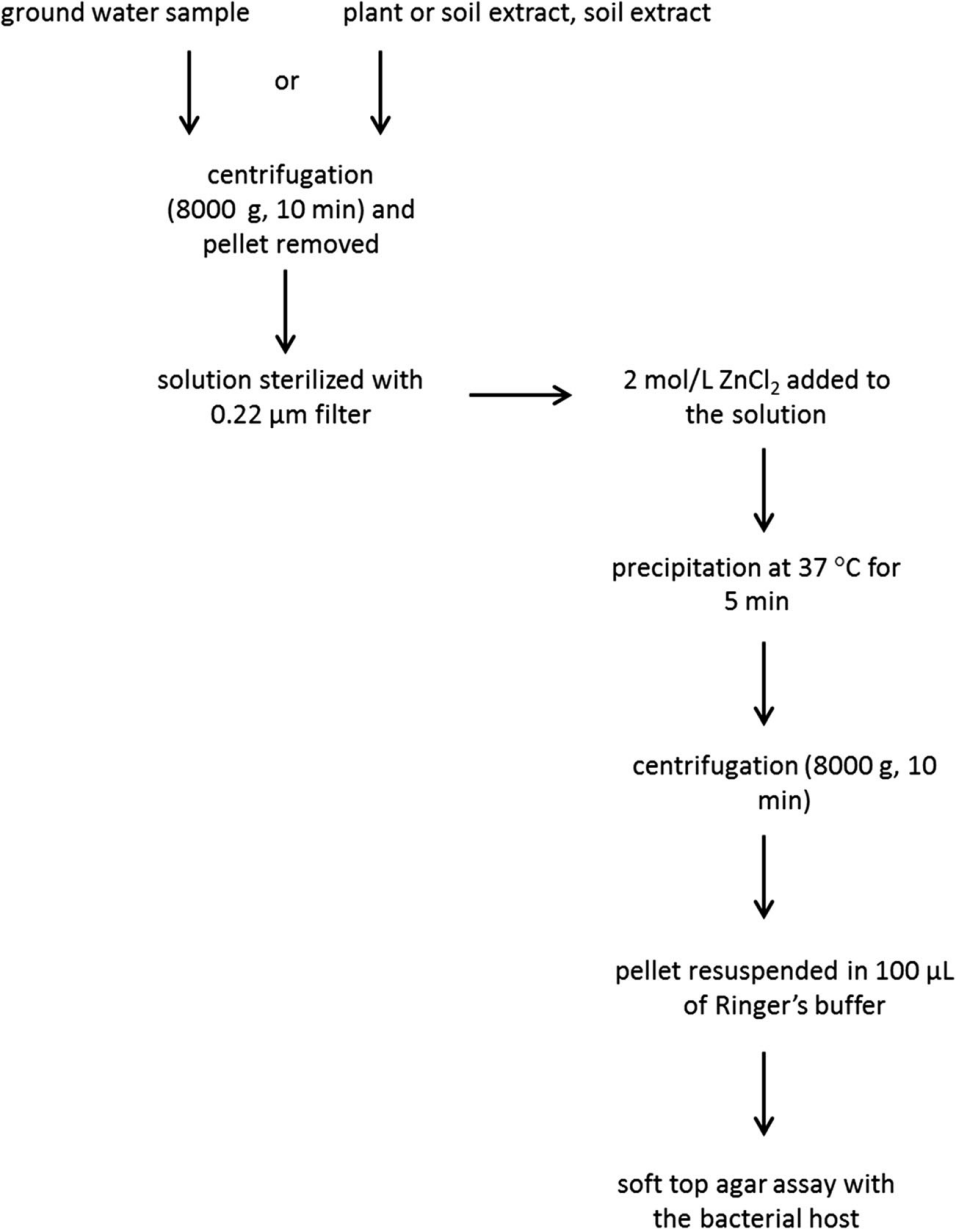
The scheme of the procedure for concentrating bacteriophages from environmental samples using precipitation with 2 mol/L ZnCl_2_.

For ZnCl_2_-based bacteriophage precipitation (Fig. 1), the phage preparations were prepared as described above in solutions containing 1, 10, 100, or 1000 PFU/mL in potato tuber extract, potato stem extract, soil extract, surface water, TSB, and 1/4 Ringer’s buffer. One millimeter of each suspension was collected and 20 μL (1:50, *v/v*) of 2 mol/L ZnCl_2_ was added to each sample. Treated samples were incubated for 5 min at 37 °C, followed by centrifugation (8000 g, 10 min) to precipitate bacteriophages. The pellet containing bacteriophages were resuspended in 100 μL of 1/4 Ringer’s buffer to resuscitate phage particles and assayed for the bacteriophage presence using a soft top agar method as described above. The presence of phage plaques on bacterial lawn was determined for each treatment. Each treatment was carried out in duplicates, and the entire experiment was independently repeated four times (eight individual samples) with the same setup and the results averaged.

The adapted bacteriophage precipitation method with zinc chloride and the enrichment method were also compared for their relative ability to detect lytic bacteriophages against pectinolytic *D. solani*, *P. carotovorum* subsp. *carotovorum*, and *P. atrosepticum* in environmental samples. For this, 191 samples of potato tuber, potato stem, soil and surface water collected in different regions in Poland in 2012–2013 and obtained from the Main Inspectorate of Plant Health and Seed Inspection, Poland were assayed as described above. The experiment was independently repeated twice and the results averaged.

## Results and discussion

To our knowledge, this study is the first report describing a simple method for direct isolation of intact, lytic bacteriophages against *Pectobacterium* spp. and *Dickeya* spp. from environmental samples bypassing the need for prior enrichment. We propose it as a method of choice to isolate viable bacteriophages against *Pectobacterium* spp. and *Dickeya* spp. bacteria from complex environmental samples.

The mode of action of phage precipitation with ZnCl_2_ has a lot in common to the salting out of proteins in protein precipitation assays (Arakawa and Timasheff 1984). Bacteriophage particles are made up mostly of a coat of proteins (head and tail proteins) around genetic material. Therefore, when in solutions containing high salt content, they will precipitate due to the electrolyte-nonelectrolyte interaction in which the nonelectrolyte (phage particles) would be less soluble at high salt (ZnCl_2_) concentration.

The newly adapted for bacteriophage ecological studies precipitation method allowed isolation of bacteriophages from samples containing significantly lower concentrations of bacteriophage particles compared to enrichment technique. We were able to isolate bacteriophages from extracts spiked with bacteriophages solutions containing as little as 1 and 10 PFU/mL with tuber or soil extract, respectively (Table 1). In contrast, with the enrichment method, we were able to isolate bacteriophages from solutions containing 10–100 PFU/mL bacteriophages. The sensitivity of bacteriophage detection with the precipitation method was therefore 10–100 times higher.

**Table 1:** Comparison of sensitivity of ϕD5 bacteriophage precipitation with ZnCl_2_ with sensitivity of ϕD5 phage enrichment in *D. solani* IPO2222 host culture.

PFU/ml	Precipitation with ZnCl_2_						Enrichment in *D. solani* IPO2222					
	Ringer’s buffer	TSB	Soil extract	Tuber extract	Stem extract	Water	Ringer’s buffer	TSB	Soil extract	Tuber extract	Stem extract	Water
1	+^a^	+	−^b^	+	+	+	−	−	−	−	−	−
10	+	+	+	+	+	+	+	+	−	−	−	+
100	+	+	+	+	+	+	+	+	+	+	+	+
1000	+	+	+	+	+	+	+	+	+	+	+	+

^a^(+) indicates the presence of plaques on bacterial lawn

^b^(−) indicates the absence of plagues on bacterial lawn

The adapted ZnCl_2_-based precipitation also allowed us to isolate bacteriophages more rapidly from a greater number of environmental samples. We screened 191 samples containing potato tubers, potato stems, and soil collected in different regions in Poland together with water samples obtained from the Main Inspectorate of Plant Health and Seed Inspection, Poland and tested them for presence of bacteriophages against three different species of SRE: *D. solani*, *P. carotovorum* subsp. *carotovorum*, and *P. atrosepticum*. The presence of lytic bacteriophages with the precipitation method was confirmed for 23 samples, whereas only four samples were positive with the enrichment method (Table 2). What is the most important; the precipitated bacteriophages after resuscitation were found to be infectious indicating that the procedure did not affect their viability.

**Table 2:** Comparison of effectiveness of bacteriophage isolation with the use of ZnCl_2_ precipitation method and enrichment of putative bacteriophages in their bacterial host cultures. Samples negative for phage presence tested with both precipitation and enrichment are not shown.

No.	Sample type	Precipitation with ZnCl_2_	Estimation of initial bacteriophage numbers after precipitation ^b^	Enrichment in bacterial host culture^c^
1	Tuber	+^a^	5	+
2	Stem	+	23	+
3	Stem	+	12	−^d^
4	Stem	+	16	+
5	Tuber	+	71	−
6	Soil	+	4	−
7	Stem	+	13	+
8	Tuber	+	16	−
9	Tuber	+	13	−
10	Soil	+	14	−
11	Stem	+	97	−
12	Tuber	+	64	−
13	Soil	+	58	−
14	Soil	+	45	−
15	Stem	+	15	−
16	Stem	+	36	−
17	Stem	+	37	−
18	Stem	+	67	−
19	Tuber	+	36	−
20	Stem	+	45	−
21	Soil	+	62	−
22	Soil	+	1	−
23	Tuber	+	11	−

^a^ (+) indicates the presence of plaques on bacterial lawn

^b^Number of phage plagues was estimated using soft top agar assay as described in (Czajkowski et al. 2013), with the assumption that each individual plague is formed by one bacteriophage particle

^c^Sample was considered as positive if bacteriophages against at least one tested bacterial species were present

^d^(−) indicates the absence of plagues on bacterial lawn

## Conclusions

In conclusion, the newly adapted method for purification of phage particles with the use of ZnCl_2_ is a cheap and faster alternative to the other methods used for detection and isolation of viable *Pectobacterium* and *Dickeya* spp. phages from the environment. It does neither require special laboratory equipment nor any special chemicals and procedures. Additionally, the new method requires considerable less time than the enrichment ones. Precipitation of phages by ZnCl_2_ does not result in a loss of the ability of bacteriophages to infect their hosts. The most important feature of this method is that precipitation procedure has a better resolution, i.e., higher sensitivity than the most-widely used enrichment procedures and hence may be used to find new bacteriophages in the environment.
